# Strategy for the Development of a Smart NDVI Camera System for Outdoor Plant Detection and Agricultural Embedded Systems

**DOI:** 10.3390/s130201523

**Published:** 2013-01-24

**Authors:** Volker Dworak, Joern Selbeck, Karl-Heinz Dammer, Matthias Hoffmann, Ali Akbar Zarezadeh, Christophe Bobda

**Affiliations:** 1 Leibniz Institute for Agricultural Engineering Potsdam-Bornim (reg. Assoc.), Max-Eyth-Allee 100, 14469 Potsdam, Germany; E-Mails: jselbeck@atb-potsdam.de (J.S.); kdammer@atb-potsdam.de (K.-H.D.); mhoffmann@atb-potsdam.de (M.H.); akzare@atb-potsdam.de (A.A.Z.); 2 CSCE Department, University of Arkansas, JBHT-CSCE 515, Fayetteville, AR 72701, USA; E-Mail: cbobda@uark.edu

**Keywords:** smart camera, NDVI, image processing, plant sensor, embedded system

## Abstract

The application of (smart) cameras for process control, mapping, and advanced imaging in agriculture has become an element of precision farming that facilitates the conservation of fertilizer, pesticides, and machine time. This technique additionally reduces the amount of energy required in terms of fuel. Although research activities have increased in this field, high camera prices reflect low adaptation to applications in all fields of agriculture. Smart, low-cost cameras adapted for agricultural applications can overcome this drawback. The normalized difference vegetation index (NDVI) for each image pixel is an applicable algorithm to discriminate plant information from the soil background enabled by a large difference in the reflectance between the near infrared (NIR) and the red channel optical frequency band. Two aligned charge coupled device (CCD) chips for the red and NIR channel are typically used, but they are expensive because of the precise optical alignment required. Therefore, much attention has been given to the development of alternative camera designs. In this study, the advantage of a smart one-chip camera design with NDVI image performance is demonstrated in terms of low cost and simplified design. The required assembly and pixel modifications are described, and new algorithms for establishing an enhanced NDVI image quality for data processing are discussed.

## 1. Introduction

Agriculture research activities focus on reducing carbon dioxide, environmental impact and cost. Therefore, precision farming, which combines various information or databases to increase the agricultural input-to-output ratio, is often used [1–3]. Plant numbers, coverage levels or the amount of biomass [4] is typically determined using sensors, for e.g., control of an online field sprayer. For online control, the sensor has to be mounted on a tractor or a self-propelled vehicle, and data processing must be available and performed rapidly to control the spray dose [5]. The coverage level or plant counts are typical values used to control a field sprayer. The coverage level can be calculated from the NDVI image of the local field conditions [2], and the plant number in a local scene can be estimated using an additional algorithm. The NDVI is a parameter used to separate vital plant pixels from soil pixels in an image or to separate vital from non-vital plants. The NIR reflection is high for vital plants and low for soil; plants absorb more light with red wavelengths, from 620 nm to 660 nm, than soil [6,7]. Nutrient supply of the plants influences absorption through chlorophyll activity in the transition band from red to NIR (660 nm to 740 nm) and thereby corresponds to the stress of the plant [8]. Reflections in the wavelength spectrum below 740 nm are higher for plants than for soil. Hence, NIR wavebands below 780 nm are commonly used for the NDVI. Thus, the difference between NIR and red is high for plants. High-quality plant cameras, or NDVI cameras, use two or more CCD chips; for example, the DuncanTech camera types MS2100 and MS3100 have adjusted pixel positions. The optical path must be compensated and aligned for this adjustment of pixel positions because the optical path of the wavelengths is deflection dependent. In comparison, the single-chip design for the NDVI camera does not need that complex optical design, but requires a special adapted double band pass filter for red and NIR bands. Rabatel *et al.* [9] showed the principal access to the NDVI with standard cameras combined with individual band pass filters for the red band and NIR band. Therefore, research interests have increased concerning the structure and design of single-chip NDVI cameras. For reasons of cost, Ritchie *et al.* [10] exchanged the optical filter in a consumer camera and observed that exposure compensation is required. The simple application of NDVI for plant detection is not as beneficial as expected; therefore, precise camera control and additional enhancement of the NDVI are needed. Langner *et al.* [11] obtained better results in the Difference Index with Red Threshold (DIRT). Evans *et al.* [12] enhanced the camera setup using a tunable liquid crystal to establish NDVI and red edge measurements.

In this study, a one-chip, low-cost sensor (USB uEye LE camera, type UI-1226LE, from IDS with a price of about 230€ (Imaging Development Systems GmbH, Obersulm, Germany) was used to examine this approach to provide a new NDVI smart camera setup compared with a multichip camera (3-chip CCD camera, type MS2100 from DuncanTech Company, Redlake Inc., San Diego, CA, USA). The disadvantages of a standard NDVI were shown and the results of an advanced NDVI algorithm were demonstrated.

## 2. Materials and Methods

The following section describes the NDVI and its application as a plant camera. First of all, we demonstrate the quality of an expensive (15k€) multispectral plant camera. For the second, low-cost, single-chip camera, algorithm modifications are required to apply NDVI functionality to the camera. The low-cost sensor without an IR-cut filter was combined with an optical low-pass filter at 645 nm (RG645, SCHOTT AG, 55122 Mainz, Germany) or a custom-made double band pass filter (ET620_60bp_780_900bp from Chroma Technology GmbH (Olching, Germany). The low-pass filter passes wavelengths higher than 645 nm (lower energy) and blocks wavelengths below 645 nm (higher energy). The dual band passes wavelengths of 620 to 660 nm and 780 to 900 nm. This reassembly enabled the formerly blocked NIR sensitivity of the chip and disabled the blue and green sensitivity in the visible band. With this change, the blue and green channels only measured the NIR intensity, and the red channel measured the sum of the red and NIR signal intensities. The signal combination in the red pixels is different from that of the commercial NDVI cameras, such as the XNite Canon 450 NDVI, which has no red signal and uses the blue and NIR channels to accommodate NDVI signals. The combination presents a disadvantage in terms of pixel saturation but is an acceptable compromise for the simplified setup. Using a camera with a digital image sensor, all camera parameters can be set using software programs, and the camera adjustments can be performed to optimize the NDVI application but not the color image. Therefore, the camera used must have disabled automatic white balancing because this process is useless after optical filter modification. Similarly, the automatic gain and exposure control must also be disabled. The typical auto-adjustment is often optimized for green color and ignores saturation in the red channel, which must be considered because of the change in the radiation intensity in the former “green” channel. With respect to the Bayer pattern and the full pixel gain control, the dynamic range for the new NIR and red channel can be adjusted. With an adapted formula, a similar NDVI can be calculated from the raw Bayer pixel intensities, considering that the debayering or demosaicing is also disabled. These results were discussed, and the disadvantages of a standard NDVI were shown. The results demonstrated the need for an advanced NDVI algorithm, and two examples with simplified algorithms for embedded systems were shown.

### 2.1. NDVI

**Figure 1.**
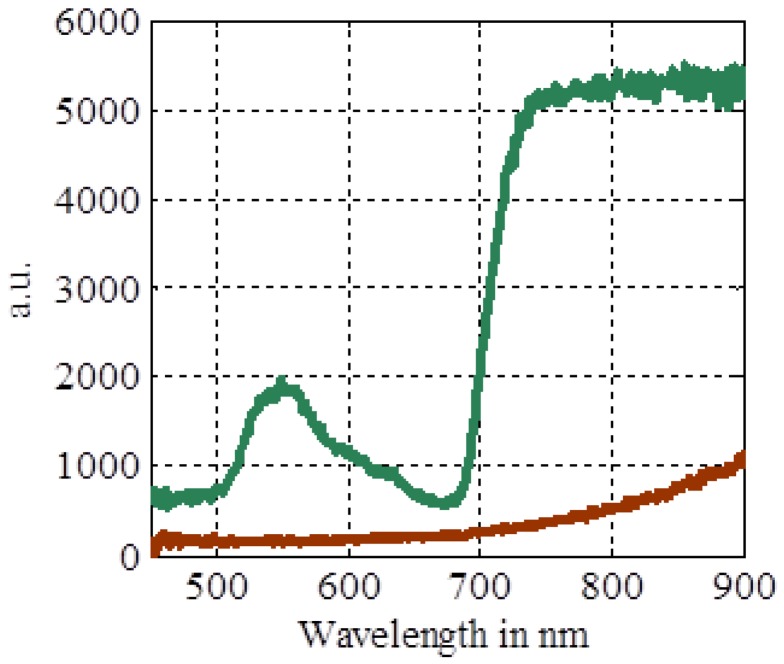
Reflectance spectral response from *Arabidopsis* (green) and organic garden soil (brown) (measured July 2010).

The NDVI facilitates the discrimination of plant from soil pixels in a digital camera and can be used in a quantitative manner to obtain information concerning the chlorophyll activity in the plant. This can be used for further analysis and cannot be performed simply using the green channel of an RGB camera. The NDVI works because of the high absorption in the red band by chlorophyll molecules and the increased reflection in the NIR band. Figure 1 shows the difference between a typical soil spectrum and the plant spectrum.

To obtain the signal difference independent from the intensities, the signal is normalized by the sum of the red channel and the NIR channel to calculate the NDVI, Rouse *et al.* [13] cited by Jones *et al.* [14]:

(1) NDVI=(NIR−R)/(NIR+R)

### 2.2. Multispectral Plant Camera

The NDVI was used in a multispectral camera (3-chip CCD camera), type MS2100, to discriminate the plant material from the soil background. This custom-made camera measures the reflection intensities in the red, infrared, and green wavelengths and is the main component of a machine vision system for the sensor-based recording of weeds [15].

**Figure 2.**
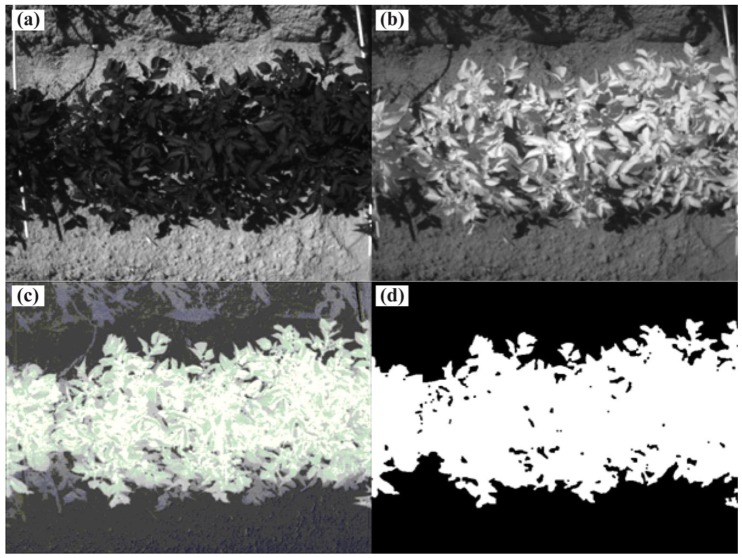
Potato plants detected using a multispectral camera. The red channel (**a**), and the NIR channel (**b**) were used to calculate the NDVI (**c**) and the binary images (**d**) through thresholding.

The camera was controlled using the custom-made control software “DT”, and the picture size is 659 H × 494 V. Image processing conducted using specialized software (SYMACON GmbH, Barleben, Germany) and includes the erosion of single pixels. Image processing and control software were run on a dust-proofed industrial computer, such as an IPC-r 4 HE (PK Computer GmbH, Eppstein, Germany).To detect the plant coverage level, only the red (peak wavelength: 670 nm, band pass size: 22 nm) and infrared (peak wavelength: 800 nm, band pass size: 65 nm) reflectance intensities of this 3 chip camera is used. Figure 2(a,b) show examples of red and NIR images of potato plants and soil.

From these images, a pixel-wise NDVI image can be calculated. Figure 2(c) shows the results, and all of the plant material shows high intensity levels; the shadow regions on the soil are at the middle level, and the soil is dark. However, the intensity continually increases from the dark soil to the bright plant levels. Therefore, the selection of the correct manual threshold for the binary image is not trivial. An enhanced NDVI or a different algorithm, such as the color angle, could overcome this difficulty. This situation is critical for the plants in the shadow region, reflecting our need to establish an enhanced NDVI for our camera design. The binary image can then be used to measure the plant coverage level or, with the use of image possessing, the amount of plants or leaves.

### 2.3. Single-Chip Plant Camera

To design a single-chip plant camera, which is useful for agricultural applications, the image quality under the worst working conditions has to be considered. To obtain this level of quality, different algorithms for a multispectral approach are required with respect to the RGB chip design. In addition, the overall path from luminance to a digital image is not straightforward [16].

#### 2.3.1. Spectral Response of the Single Chip

**Figure 3.**
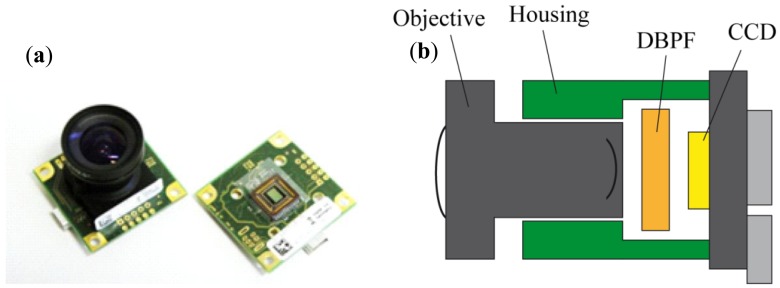
(**a**) IDS uEye camera: USB UI-1226LE-M/-C and (**b**) the outline for the setup with the implemented double band pass filter (DBPF).

To evaluate the possibility of an acceptable single-chip plant camera, we used an IDS camera (Figure 3) with an Aptina (Aptina Imaging Corporation, San Jose, CA, USA) chip MT9V032STC CMOS image sensor type with 752 H × 480 V pixels.

**Figure 4.**
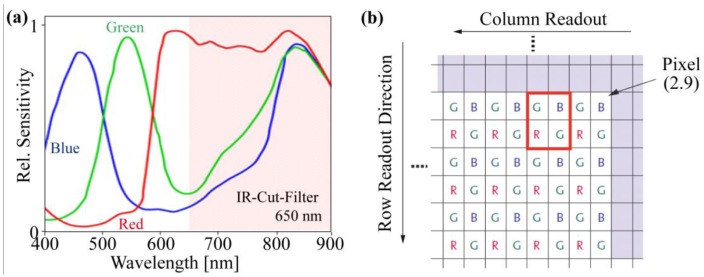
(**a**) The spectral response of the MT9V032STC CMOS image sensor from the IDS datasheet and (**b**) the Bayer pattern from the Aptina datasheet.

**Figure 5.**
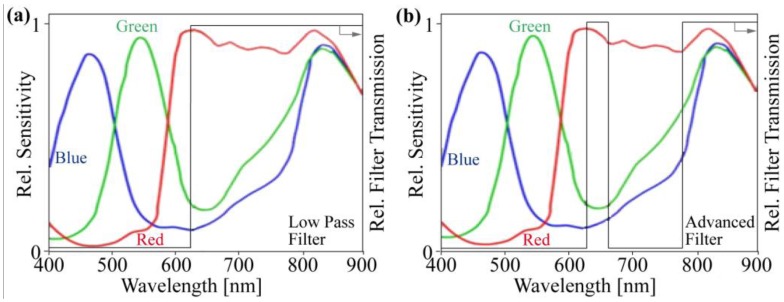
(**a**) Enabling the RGB CMOS chip for red and NIR sensitivity through ideal low-pass (**b**) or ideal double band pass filters.

This sensor type has the advantage that after disabling the IR-Cut-Filter, all pixels are sensitive in the NIR regime (Figure 4(a)), which is required for good RGB images. For this camera type, the IR-Cut-Filter can be removed after opening the lens holder housing. Instead of the IR-Cut-Filter (Figure 4(a)), a low-pass filter enables NIR sensitivity and disables the green and blue sensitivity of the chip (Figure 5(a)).

This single RGB chip has an asymmetric color pattern. The green pixels are represented twofold more than the red and blue pixels (Figure 4(b)). Therefore, the “green” precision is better, with respect to the increased sensitivity of the human eye for green color. The automatic gain control of the camera varies the output levels of the color bands, but often and in our case, this feature is optimized for the green channel and not for the red. However, in the following paragraph, we explain why the automatic gain control is useless and must be disabled for plant cameras.

#### 2.3.2. Filter Design for the Single-Chip Plant Camera

**Figure 6.**
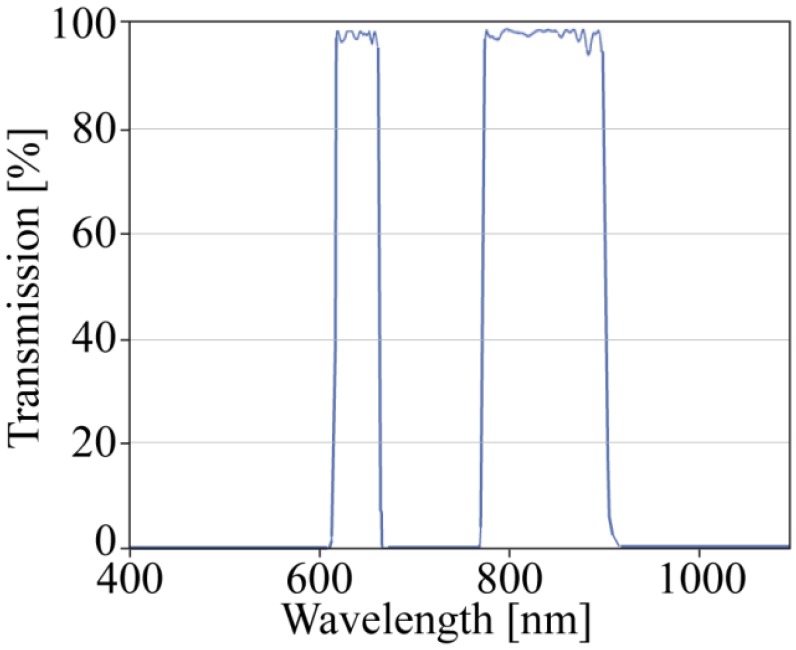
Filter design of the custom-made ET620_60 bp_780_900 bp dual band pass filter from Chroma.

By implementing a low-pass filter, the blue and green sensitivity is disabled, and these pixels are now sensitive in the NIR band. The red channel remains sensitive in both the red and the NIR bands. For a more precise separation between the NIR and red channels, we integrated a customized enhanced double band pass filter for the red and NIR bands (Figures 5(b) and 6). This enhanced filter eliminates the transient region between red and NIR, and the output response is more defined in terms of which wavelengths are responsible.

**Figure 7.**
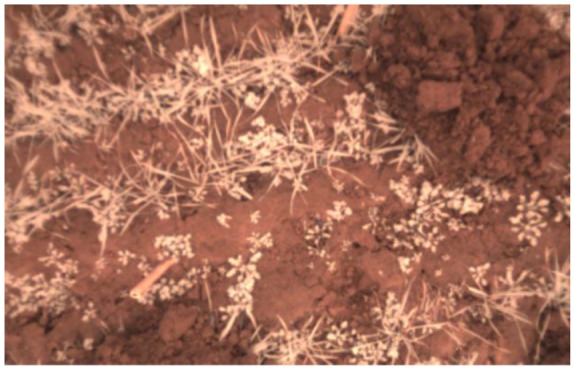
False color image of the modified camera. Weed in winter wheat is shown. The drilling distance was 14 cm and the shutter time was 250 μs.

With the modification of the optical path, the image sensor can be used as an NDVI plant camera. A typical false color output image is depicted in Figure 7, with a typical red and white view.

The red channel dominates because of the sensitivity in both bands. White is the second most dominating color because the NIR sensitivity is nearly equal for all color channels and R = G = B = 1 is translated into white for an image. As a result, the plants appear white in this image. However, this image was captured under stray light weather conditions and not under direct sunlight, which becomes more difficult, as discussed later.

#### 2.3.3. Demosaicing and Debayering

The Bayer pattern for this image sensor is shown in Figure 4(b), and at each pixel position, only single color information is detectable. Therefore, most standard single-chip RGB cameras use an interpolation algorithm to complete the missing color information at each pixel. There are many different algorithms for demosaicing or debayering the raw image matrix, which enhances the image impression, solving the problem of missing color information under a special scene assumption: a natural image is being used, and the image sensor resolution is higher than the resolution of the objective, with an overlay between the pixels.

**Figure 8.**
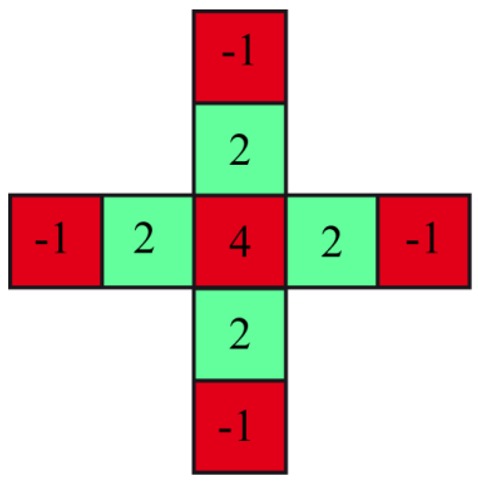
Example of a demosaicing pattern and coefficients corresponding to Malvar *et al.* [17] from Microsoft Research.

Both assumptions do not fit the scheme of our plant camera. The camera does not detect natural images after implementing the additional optical filter, and the pixel resolution is low compared with the objective for megapixel cameras. The demosaicing filter example in Figure 8 shows that the contrast or difference between the red and green channels, which will be used for the NDVI, will be filtered out and is therefore useless for our application and must be disabled. If a mismatch between the pixel positions is acceptable, one quadruple, such as the red square in Figure 4(b), can be combined with one NDVI pixel. Otherwise, a simple linear interpolation for each individual color channel can also be used.

#### 2.3.4. Adapted NDVI for A Single-Chip Plant Camera

The different signal content in the single-chip plant camera compared with the multi-chip camera generates a different algorithm for the NDVI. The red channel (R) includes the NIR plus red intensities and the blue (B) and green (G) channels have only the NIR signal content (Figure 5(b)):

(2) $$\text{NDVI}=\frac{\text{NIR}-R}{\text{NIR}+R}\to {\text{NDVI}}_{\text{CMOS}}=\frac{(B+G)-R}{R}$$

After calibrating with red and NIR light sources, a gain correction factor can be applied to the “blue” and “green” channel. The advantage of the selected image sensor is the nearby identical signal sensitivity for all color channels in the 850 nm regime. Therefore, for our first implementation of the NDVI in the PC software, the gain calibration was not used.

#### 2.3.5. Gain Control

For establishing an NDVI or plant camera, the gain control is an important factor because pixel saturation must be prevented. A saturated pixel generates incorrect results for the NDVI calculation and does not appear as simply a bright pixel. The chip integrated automatic gain control in our camera is optimized for the green color, which is the dominant color for the human eye. For the NDVI_CMOS_, the red channel tends to be saturated because of the combined intensities from the red and NIR wavebands. To prevent pixel saturation, some camera chips generate a histogram together with the image; otherwise the histogram must be calculated. With respect to the highest pixel intensity, an automatic gain control feature can be implemented. The camera should be started with the same procedure as a successive approximation register analog-to-digital converter works. The camera starts with his middle gain setting. If there are pixels at the highest bit value, the gain will be reduced by half of the range. Otherwise, the camera will obtain more gain from 1/2 to 3/4 of the maximum gain. Then, the procedure starts again, and after eight steps, the correct gain value is detected with a precision of eight bits. After reaching a high-level frame rate, the camera requires only small gain variations between the images because the image itself can vary in small increments. Regarding the prevention of pixel saturation, the gain reduction will be initialized immediately, and gain rising will be initialized after the pixel intensities are reduced under a defined threshold. Thus, hysteresis will be implemented to reduce small-level oscillations. The camera is connected via USB port to the PC. With the use of the activeX software interface from Microsoft the gain and the shutter time can be controlled manually or automatically by a designed program in the programming language Matlab. In most cases, the camera chips have more than one gain stage and the first gain should be at the highest level to optimize the signal-to-noise ratio of the chip.

### 2.4. Measurement Conditions

To describe the difficulties of an NDVI calculated from a single-chip plant camera under outdoor conditions, the best and the worst lighting conditions are described in this paragraph. The best weather conditions are observed on a homogeneous cloudy day, when only stray light exists. The stray light produces an image in which there are no shadow regions, and the overall dynamic range is reduced. The worst condition is bright angular direct sunlight because the shadow regions have more than ten times less intensity, and therefore a much higher dynamic range is required. In addition, the quantitative NDVI value changes from the shadow to the sunlight region because the ratio of NIR to red changes for atmospheric scattered light, which lights the shadow region. Scattering is dependent on a fourth power of the wavelength. NIR light is less scattered and has less intensity in the shadow regions, but when the shadow passes through a leaf or the plant itself, the opposite situation is presented because NIR light has a higher transmission than the absorbed red light. Therefore, small intensities of an unknown combination of atmospheric scattered light and plant-transmitted light exist in the shadow region. Considering the normed index, these effects could also reflect the fact that soil shadow regions are brighter than soil sun regions.

## 3. Results and Discussion

**Figure 9.**
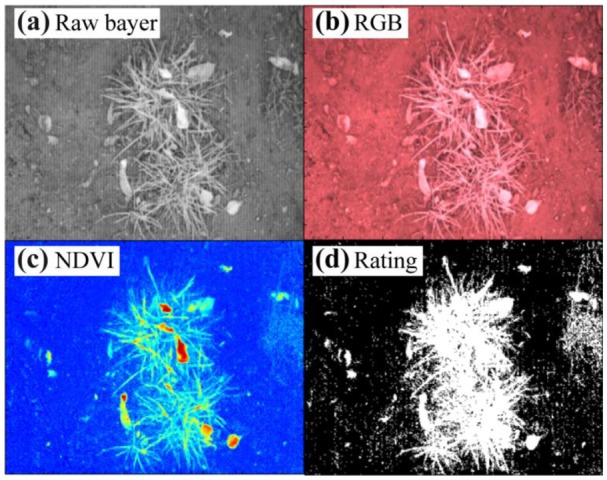
(**a**) Raw image of tufts of grass and leafs added by wind, (**b**) false color image, (**c**) NDVI image in rainbow colors from blue (zero) to red (256), and (**d**) binary image under stray light conditions and with a shutter time of 250 μs.

**Figure 10.**
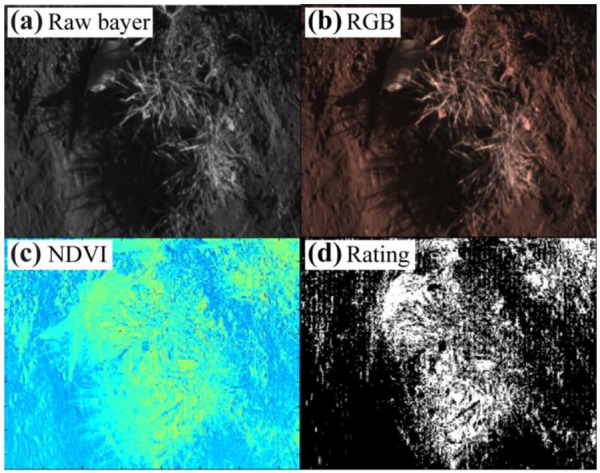
(**a**) Raw image of tufts of grass and leafs added by wind, (**b**) false color image, (**c**) NDVI image in rainbow colors from blue (zero) to red (256), and (**d**) binary image under direct angular sunlight and a shutter time of 90 μs.

Demonstrating the usability of the single chip plant camera small sets of images under different lighting conditions and with different plants were taken. The results shown below in Figures 9 and 10 illustrate the difficulties of the single-chip plant camera. These results demonstrate the need for an adapted NDVI algorithm to establish a useful plant camera.

Figure 9 shows that the NDVI image under stray light conditions looks fine, but it is difficult to obtain the correct manual threshold for the binary image. A higher contrast between the plants and the soil background would simplify the threshold estimation or calculation using adapted algorithms.

The most difficult conditions under angular sunlight are shown in Figure 10, demonstrating that there is nearly no contrast between the plants and the soil in the shadow regions, and therefore, the binary image includes incorrect classifications in the shadow region. Thus, both (all) NDVI images need some enhancement in the algorithms to overcome these drawbacks. The difference between minimum and maximum values for the pixel intensities provides information concerning the dynamic range and the lighting conditions on the field. Notably, it is difficult to obtain similar desired results for the stray light and the direct sunlight conditions. The following difficulties need to be accounted to establish a single chip plant camera:

- The NDVI_CMOS_ could be faster corrupted by pixel saturation, because of the sensitivity of the R channel for red and NIR light.
- Under direct sunlight conditions the threshold level differs in the shadow region of the image and wrong classifications results out of it.
- It is difficult to apply the adequate threshold level even under best stray light illumination condition, because the cross-section of soil and plants overlap for the NDVI values.
- The enhanced algorithms for the solution should be plain to establish those in small embedded systems for online control applications.

### 3.1. Embedded System for A Single-Chip Camera

Although Equation (1) for the NDVI appears to be simple, a significant amount of calculation power is required for an embedded system with a small processing unit, such as a microcontroller with a 100 MHz clock. Whereat the actual prototype uses a PC for the calculations, this is not a low cost solution for an embedded system. All used formulas should be minimized in terms of calculation power for the embedded system, especially when additional functionality must be included. A future version should use an automatic gain control function to establish a more or less stable intensity, if the illumination control and a reference background are available. If this application causes a stable denominator (NIR + red) the calculation power intensive division for normalization is not necessary. Alternatively, modern microcontrollers have DSP functionality (digital signal processor) in an included coprocessor, such as the Cortex M4F by ARM, or multiprocessor chips with DSP cores, such as the OMAPTM5 by Texas Instruments.

### 3.2. Enhanced NDVI_CMOS_ Algorithm for A Single-Chip Camera Used Under Stray Light Conditions

With the knowledge that plants have high intensities in the NIR band under all lighting conditions, it should be possible to amplify the plant soil contrast using the NIR color channel pixel information. For the enhanced NDVI_CMOS_ (eNDVI_CMOS_) Equation (2) is extended so that all of the pixel results will be multiplied by the average of the pure NIR color pixels B and G:

(3) $${\text{eNDVI}}_{\text{CMOS}}=\frac{(B+G)-R}{R}\cdot \left\{\frac{B+G}{2}\right\}$$

**Figure 11.**
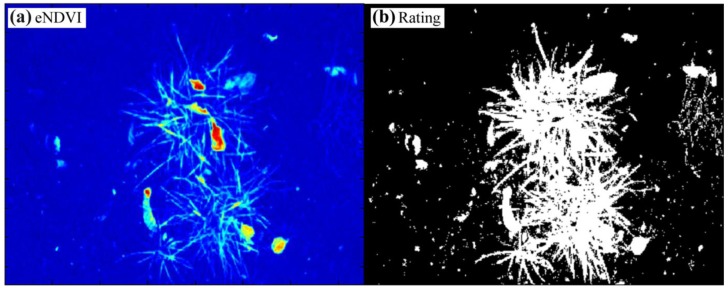
(**a**) Image of the eNDVI_CMOS_ with the raw data from Figure 9 in rainbow colors from blue (zero) to red (256) and (**b**) the resulting binary image.

The applied gain proportional to the NIR intensities results in a reduction in the intensity level for the soil pixel, even in the inner regions of the two tufts of grass in the center of the image (Figure 11(a)). A threshold for the correct rating was established, and only a few soil pixels were wrongly classified (Figure 11(b)). Single-pixel deletions or a five-point Gaussian filter will eliminate most of the wrong pixels. The ratio of the white to black pixels corresponds with the coverage level, and this correlation can be directly used to control the spray dose rate of a field sprayer. The result can also be used to mask additional feature extractions, such as shape or contour, or to analyze the NDVI plant information in a quantitative manner. Thus, the algorithms for eNDVI_CMOS_ are simple and can easily be installed on small microcontroller or field programmable gate array (FPGA) boards. However, this situation can change under direct sunlight conditions, where the eNDVI_CMOS_ cannot efficiently separate the plants from the soil in the shadow regions of the image.

### 3.3. Range-Extended NDVI for Single-Chip Plant Cameras Under Direct Sunlight Conditions

Under direct sunlight conditions, the dynamic range of the pixel intensities is much higher, and the spectral conditions for the illumination also change for the shadow region, as described above. Thus, we could not calculate a threshold level that works for both areas. The shadow region was consistently underrepresented using thresholds for the bright sunlight regions. Therefore, a nonlinear gain function is needed and was derived from examined images. The Equation (3) is extended by the nonlinear function *f*(*x*), in which x represents the pixel intensity from zero to one:

(4) $${\text{reNDVI}}_{\text{CMOS}}=\frac{(B+G)-R}{R}\cdot \left\{\frac{B+G}{2}\right\}\cdot f(x)|\;\begin{array}{l} f(x)=1;\\ f(x)=3-10\cdot x;\\ f(x)=2; \end{array}\begin{array}{c} 0.2<x<1.0\\ 0.1<x\leq 0.2\\ 0.0\leq x<0.1 \end{array}$$

The range-extended NDVI_CMOS_ (reNDVI_CMOS_) could overcome this problem because the low level pixels are amplified in the shadow region. The formula was empirically determined out of several normalized images and it enhances images for all illumination conditions. Whereas, thresholding is always critical for NDVI values (Table 1).

For the shadow soil region the NDVI is overrepresented and for the reNDVI_CMOS_ a threshold of 0.1 is almost reasonable. Applying the range-extended NDVI_CMOS_ algorithm to the image in Figure 10(c) generated the result shown in Figure 13.

**Figure 12.**
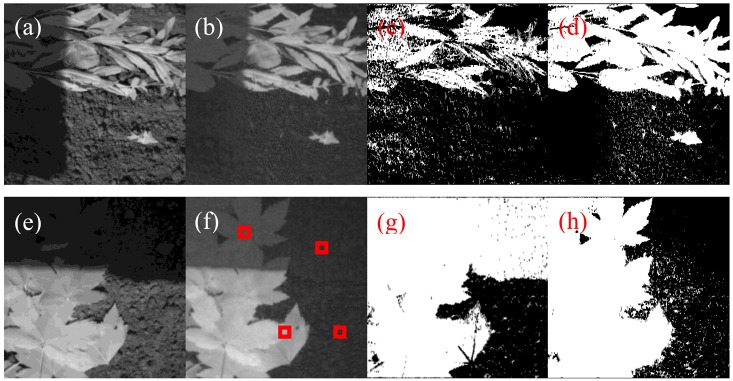
(**a**) Raw image of a high dynamic range situation of soil covered piecewise by leafs, (**b**) the applied reNDVI, (**c**) binary image of the NDVI, (**d**) binary image of the reNDVI, (**e**) raw image of a high dynamic range situation of soil covered piecewise by leafs, (**f**) the applied reNDVI, (**g**) binary image of the NDVI, and (**h**) binary image of the reNDVI. All binary images are five-point Gaussian filtered results.

**Figure 13.**
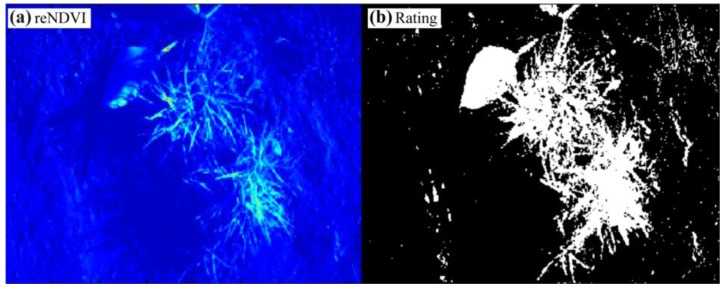
(**a**) Image of the applied reNDVI with the raw data from Figure 10 in rainbow colors from blue (zero) to red (256) and (**b**) the five-point Gaussian filtered binary result.

**Figure 14.**
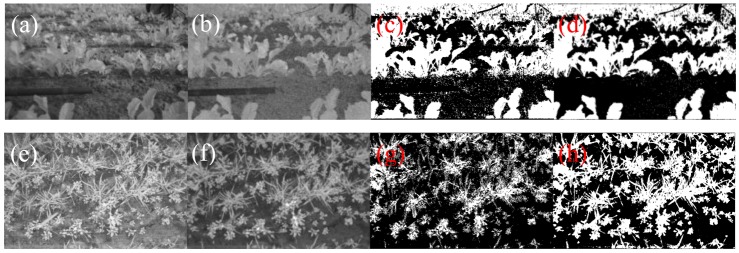
(**a**) Raw image of a salad field (by courtesy of Cruse Leppelmann Kognitionstechnik GmbH), (**b**) reNDVI image, (**c**) binary image of the NDVI, (**d**) binary image of the reNDVI, (**e**) raw image of winter wheat in the early growths state by Potsdam, Germany, (**f**) reNDVI image, (**g**) binary image of the NDVI, and (**h**) binary image of the reNDVI. All binary images are five-point Gaussian filtered results.

The combination of the eNDVI and the nonlinear function solves the threshold problem of high dynamic range images. First, the higher gain for the NIR signal spreads from the plant to the soil in the critical shadow region, and in Figure 13(a), the large leaves exhibit increased brightness, even in the shadow. Second, the additional gain for all low intensities enhances this area, and the plants can be captured using one threshold for the entire image. Figure 13(b) shows the nearly ideal contour of the leaf in the binary image after applying the reNDVI. Obviously, it becomes easier to obtain a better NDVI and rating results with higher performance cameras, equipped with a higher dynamic range and less pixel noise, but obtaining acceptable results with a low-cost single-chip camera for plant detection is required for many agricultural applications. Figures 12 and 14 demonstrate the advantage of the applied algorithm. A gain control is needed to establish these results.

### 3.4. Saturation Influence of the NDVI_CMOS_ for Single-Chip Camera under Direct Sunlight Conditions

**Figure 15.**
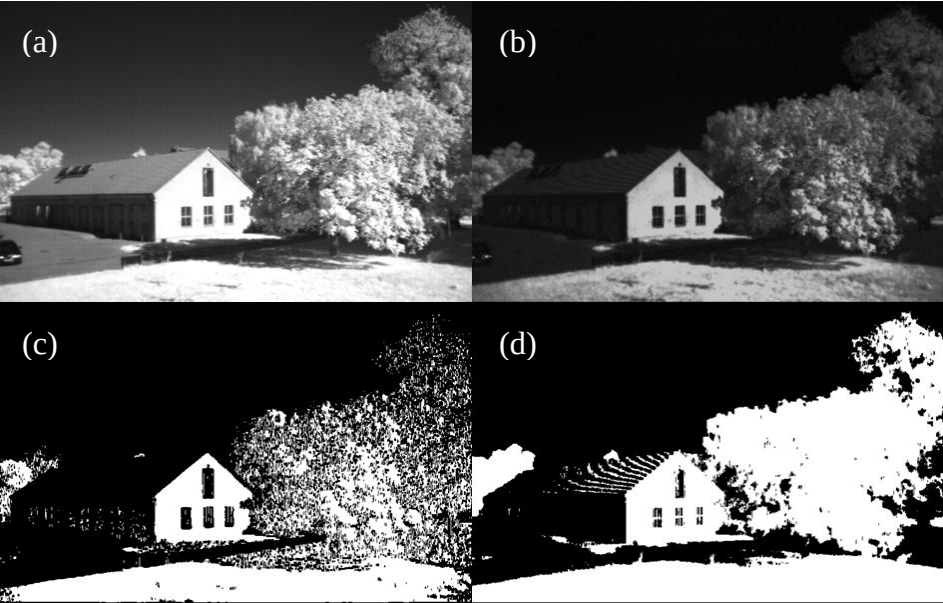
(**a**) Raw image of a landscape under direct sunlight condition, (**b**) reNDVI_CMOS_ image, (**c**) binary image for the NDVI and (**d**) binary image for the reNDVI_CMOS_. Both binary images are five-point Gaussian filtered results.

The prevention of pixel saturation is important to avoid wrong classification. As described in Sections 2.3.4 and 2.3.5 the R channel includes red and NIR intensities and is therefore saturated first. The bright building wall in the non-agricultural image in Figure 15 with saturated pixel shows the wrong classification for both NDVI and reNDVI_CMOS_ images in Figure 15(c,d). Except this, the reNDVI_CMOS_ could classify the grass area in the shadow region and the trees more precise.

## 4. Conclusions

The results demonstrate that a plant detection camera was designed with a new arrangement of the optical filter design to obtain a low-cost digital image sensor. This filter disables the blue and green color sensitivity and enables the true path of NIR light in the 850 nm regime. Therefore, the blue and green pixels can be used for new, pure NIR channels. The contrast in the NDVI image could be optimized with a more specific optical filter. Whereas a simple low-pass filter is working; a double band pass filter for the transmission of the red band, from 620 nm to 660 nm, and the NIR band, from 780 to 900 nm removes the intensities between the two transmission bands. This results in an elimination of the transient region of the NIR sensitivity of the different camera channels, and therefore the output response is better defined.

The use of the raw Bayer image of the CCD chip reduces the number of pixels; therefore, the data bus to the processing unit was not overloaded with large RGB arrays, which would occur with a tiff format. The user can decide whether the data reduction is important for implementing the algorithm in an embedded system with restricted memory and processor power resources, or whether a debayering algorithm is needed for pixel filling. Either strategy will be used equally, and the algorithm derived from the camera must be disabled because it is optimized for natural RGB images and is useless for the NDVI image. This is also true for green color optimized automatic gain control functions because the new “red” channel is the most dominating, with summed red and NIR intensities. Thus, full pixel control is indispensible for a single-chip plant camera.

Whereas the NDVI is a well-known and frequently used index for gathering the information content of plants, the direct application of a low-cost, single-chip plant camera is difficult under real outdoor conditions. Therefore, a simple threshold for detecting, for example, the coverage level is not sufficient. Even under the best lighting conditions, that is, stray light, the NDVI requires some enhancement to reach sufficient performance or precision for coverage level. For the eNDVI, we demonstrated that multiplication using the averages of both NIR channels generates a successful rating for plant soil separation. However, an even better algorithm is required under the worst illumination conditions with angular sunlight. Under angular sunlight, minimum the plant itself generates shadowed regions in the image. In these shadowed regions, it was possible to use the same threshold for distinguishing the plant from the soil. However, the plants were underrepresented using this threshold for bright areas. The establishment of the range extended NDVI could overcome this drawback, and leaves with bright areas and shadows could be efficiently separated from the soil. This could be accomplished using a nonlinear gain, which additionally amplifies the low level values and enhances the values for the regions of the plant that are in the shadow over the threshold value. The whole algorithm requires a small amount of additional calculation power and therefore can be easily installed in a small embedded system with a microcontroller or an FPGA. Because the algorithm for the eNDVI and reNDVI is currently sufficient, the next steps are the implementation of an automatic thresholding algorithm for an autonomous plant camera. The results will facilitate the prediction of a successful implementation strategy, as will be shown in future research.

## Figures and Tables

**Table 1.** NDVI_CMOS_ and reNDVI_CMOS_ results for 10 by 10 averaged pixel values in a range from 0 to 1. The regions where taken out of the red squares in Figure 12(f).

	**NDVI_CMOS_**		**reNDVI_CMOS_**	

	**Plant**	**Soil**	**Plant**	**Soil**

Shadow	0.62	0.57	0.15	0.097
Sunlight	0.47	0.31	0.34	0.087
